# Endorectal Ultrasound Shear-Wave Elastography of Complex Rectal Adenoma and Early Rectal Cancer

**DOI:** 10.3390/diagnostics12092166

**Published:** 2022-09-06

**Authors:** Martina Kastrup Loft, Malene Roland Vils Pedersen, Jan Lindebjerg, Hans Bjarke Rahr, Søren Rafael Rafaelsen

**Affiliations:** 1 Department of Radiology, Vejle Hospital, University Hospital of Southern Denmark, Beriderbakken 4, 7100 Vejle, Denmark; 2 Department of Regional Health Research, University of Southern Denmark, Campusvej 55, 5000 Odense, Denmark; 3 Danish Colorectal Cancer Center South, Vejle Hospital, University Hospital of Southern Denmark, 7100 Vejle, Denmark; 4 Department of Pathology, Vejle Hospital, University Hospital of Southern Denmark, Beriderbakken 4, 7100 Vejle, Denmark; 5 Department of Surgery, Vejle Hospital, University Hospital of Southern Denmark, Beriderbakken 4, 7100 Vejle, Denmark

**Keywords:** endorectal ultrasound, shear-wave elastography, diffusion-weighted imaging, magnetic resonance imaging, rectal cancer, rectal adenoma, stromal fraction, tumor–stroma ratio

## Abstract

Purpose: To investigate the diagnostic performance of endorectal ultrasound (ERUS), shear-wave elastography (SWE), and magnetic resonance imaging (MRI) in patients with a complex rectal adenoma or an early rectal cancer, i.e., T1 or T2 adenocarcinoma in a clinical setting, and to evaluate the association between SWE and stromal fraction (SF) and apparent diffusion coefficient (ADC) and SF. Method: This prospective study included patients undergoing ERUS and SWE for a rectal tumor subsequently confirmed by histopathology to be an adenoma or a T1 or T2 adenocarcinoma. The accuracy of the imaging methods was assessed by comparing the T category as determined by ERUS and MRI with histopathology, which served as the gold standard. SF was assessed on surgical specimens. Results: A total of 86 patients were included. Of these, 62 patients had adenomas and 24 patients had carcinomas, of which 11 were T1 tumors and 13 were T2 tumors. ERUS and MRI yielded sensitivity, specificity, and accuracy of 0.79 and 0.73, 0.95 and 0.90, and 0.86 and 0.78, respectively, for discrimination between benign and malignant lesions. The area under the receiver operating characteristics curve for SWE was 0.88, and with a cut-off value of 40 kPa the sensitivity, specificity, and accuracy were 0.79, 0.89, and 0.86, respectively. There was a positive correlation between SF and SWE with a *p*-value of <0.001 and a negative correlation between SF and ADC with a *p*-value of 0.011. Conclusion: Both ERUS and MRI classified T categories with a high accuracy; however, ERUS classified more adenomas correctly than MRI. In this small population, SWE could differentiate an adenoma from early carcinoma. SF was correlated with both SWE and ADC, as increasing SF tended to yield higher SWE and lower ADC values.

## 1. Introduction

Treatment options for complex rectal polyps and early rectal cancer encompass a variety of modalities, ranging from major surgery over local and endoscopic resection to non-operative treatment with chemoradiotherapy [1]. These options are discussed at a multidisciplinary team (MDT) meeting [2] to reach a consensus on the adequate treatment for the individual patient. The MDT assessment is based on clinical and endoscopic findings, histopathology, and—in particular—imaging. Optimal imaging is, therefore, crucial. The European Society of Gastrointestinal and Abdominal Radiology (ESGAR) guidelines recommend magnetic resonance imaging (MRI) for all patients [3], but early T categories (i.e., T1 and T2) are frequently overstaged by MRI [4,5]. The European Federation of Societies for Ultrasound in Medicine and Biology (EFSUMB) and European Society of Medical Oncology (ESMO) recommend endorectal ultrasound (ERUS) when evaluating early rectal cancer [1,6]. According to a recent review, ultrasound elastography provides information on tissue stiffness and increases the ability of ERUS to distinguish between a rectal adenoma and cancer [7]. Two elastography techniques are used in clinical practice, strain elastography (SE) and shear-wave elastography (SWE). SWE is considered to be more operator-independent than SE, as the required deformation is produced by the transducer instead of by manual compression [8]. Few studies have addressed the ability to discriminate between an adenoma and early rectal cancer, and they have only done so for SE [9,10]. Diffusion-weighted imaging (DWI) for MRI derives contrast from the differences of the motion of the water molecules within the tissue [11], and a negative correlation between SWE and apparent diffusion coefficient (ADC) has been shown for advanced rectal cancer [12]; however, the correlation has not been investigated in early stages. The molecular transformation from adenoma to adenocarcinoma includes interaction between tumor cells and stromal cells [13]. A high stromal fraction (SF) (i.e., SF ≥ 50%) is associated with tumor aggressiveness and increased tissue stiffness [14]. The association between SF and ultrasound elastography has not previously been investigated in rectal cancer.

The aims of our study were: (1) to investigate whether SWE can discriminate between complex rectal adenoma and early rectal cancer; (2) to evaluate the diagnostic performance of ERUS and MRI in a clinical setting; (3) to investigate the association between SF and SWE.

## 2. Materials and Methods

This single-center study was conducted prospectively at the Department of Radiology, Lillebaelt Hospital, Vejle, University Hospital of Southern Denmark. We consecutively included patients from 1 March 2020 to 31 December 2021. Patients were referred to the Department of Radiology for evaluation of a rectal lesion after endoscopic visualization. Inclusion criteria were: (i) patients examined by ERUS including SWE images; (ii) adult patients above 18 years of age; (iii) tumor location ≤ 15 cm from the anal verge on MRI; (iv) tumor surgically removed, endoscopically and/or by formal resection, within 30 days after ERUS evaluation; (v) histopathologically confirmed adenoma, or T1 or T2 adenocarcinoma. Exclusion criteria: (i) low image quality; (ii) previous pelvic surgery or chemoradiotherapy; (iii) patients receiving neoadjuvant therapy.

### 2.1. Endorectal Ultrasound and Shear-Wave Elastography

All patients underwent ERUS performed by one of five radiologists specialized in colorectal imaging. We used a Canon Aplio i800 (Canon Medical Systems, Otawara, Tochigi, Japan) ultrasound machine equipped with 2D SWE software and an endocavity transducer (PVT-781VTE 3.6–10 MHz) covered with a condom. All patients took an enema prior to the examination.

Patients were placed in left lateral decubitus position, and a digital rectal examination was performed to ensure the correct positioning of the transducer. Tumor T category assessed by ERUS is indicated with the prefix “u”. SWE images were obtained without applying pressure to the area. Three separate regions of interest (ROI) were selected within the tumor. The SWE imaging procedure was repeated, and a minimum of six ROIs were obtained. The radiologists were blinded to patient symptoms and histopathology but not blinded to the MR images. In order to locate the tumor, radiologists were also not blinded to tumor distance from the anal verge nor were they blinded to whether the patient was referred to an additional computed tomography scan suggesting the endoscopist’s suspicion of malignancy.

### 2.2. Magnetic Resonance Imaging

MRI with diffusion-weighted imaging (DWI) was performed prior to the ERUS procedure. We used a 3.0 Tesla MRI scanner (Phillips Healthcare, Ingenia, Best, The Netherlands) with an anterior coil. Patients were placed in prone position. The MRI scan protocol included T2-weighted sequences in coronal, sagittal, and axial plans and a diffusion-weighted sequence including five b-values ranging from 0 to 1000. Detailed MRI scan parameters are shown in Table 1. Adequate MRI angulation was performed by radiographers supervised by a dedicated abdominal radiologist [15].

Each MRI scan was reviewed, in consensus, by two of four radiologists with >10 years of imaging experience with colorectal disease. The prefix “mr” is used as indication of T category assessment by MRI. Images were viewed using the hospital’s Picture Archive Communication System (PACS) on a diagnostic screen (21.3″ Monitor CCL358i2, Totoku, JVCENWOOD Corporation, Kanagawa, Japan). Diffusion restriction was assessed using the apparent diffusion coefficient (ADC), obtained by placing one freehand ROI covering the entire tumor area. ADC was recorded in 10^−3^ mm^2^/s.

### 2.3. Histopathology

All resected specimens were analyzed by an expert pathologist specialized in colorectal disease. Tumors were classified according to the TNM classification system by the American Joint Committee on Cancer (AJCC 8th edition) [16], and the prefix “p” indicates histopathologic T classification, which serves as gold standard. Malignant tumors were classified as pT1 if they were limited to the submucosa with at least 1 mm microscopic distance to the deep resection margin, or, if this distance could not be assessed with certainty, the lesion could be macroscopically lifted and resected endoscopically in the submucosal plane, and no residual tumor was found at subsequent completion resection. SF was assessed on resected tissue of a slice from the most invasive part of the tumor. The scoring was performed at 200× magnification in the area with the highest fraction of stroma. A score below 50% was considered a low SF, and 50% or above was considered a high SF [17].

### 2.4. Statistical Analysis

A Research Electronic Data Capture (REDCap) database was used for secure collection and management of data. Data were analyzed using Stata statistical software (version 17.0, Stata Corp., College Station, TX, USA).

The diagnostic performance of ERUS and MRI was assessed by calculation of sensitivity, specificity, positive predictive value (PPV), negative predictive value (NPV), and accuracy for discrimination between benign and malignant lesions. Additionally, the correlation between pathologic T category and the category determined by ERUS and MRI were assessed using kappa. The kappa values considered were slight (0–0.20), fair (0.21–0.40), moderate (0.41–0.60), substantial (0.61–0.80), and perfect (0.81–1.00). Differences in SWE and ADC values between groups (i.e., grouped according to pathologic T category) were compared using a one-way analysis of variance (ANOVA), and the Jonckheere–Terpstra test was used for trend. To analyze the diagnostic performance of SWE, we created a receiver operating characteristic (ROC) curve and generated the area under the ROC curve (AUC). Cut-off values between adenoma and adenocarcinoma that maximized sensitivity and specificity were chosen to calculate PPV, NPV, and accuracy of SWE. *p*-values of <0.05 were considered indicators of significant difference.

### 2.5. Ethics

All patients signed a consent form after receiving oral and written information, according to the Helsinki Declaration. The study was approved by the Regional Committees on Health Research Ethics for Southern Denmark (S-20190176) and registered at ClinicalTrials.gov (NCT04409990).

## 3. Results

### 3.1. Patients

**Figure 1:**
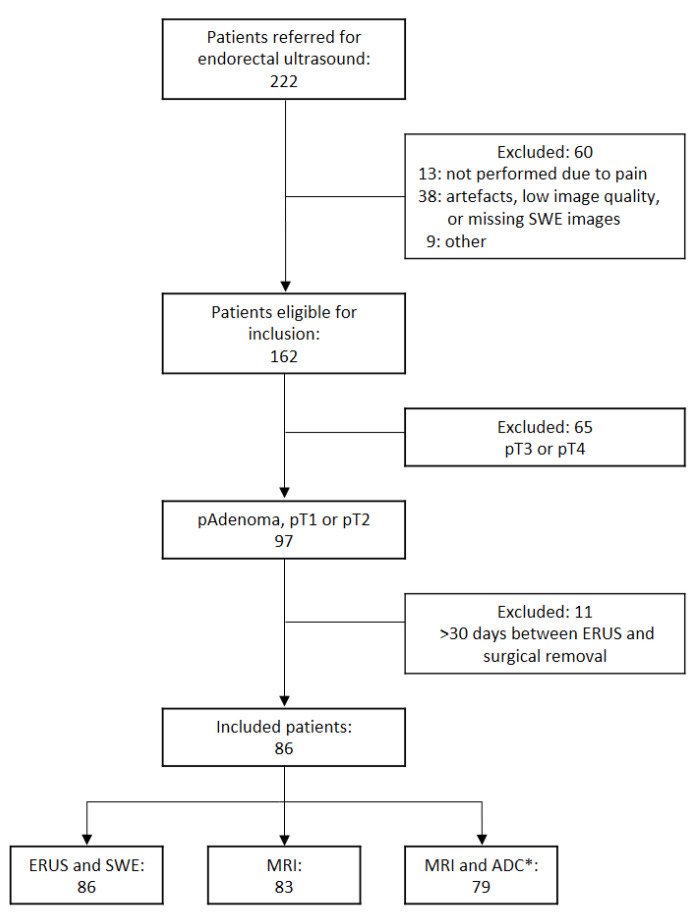
Flow chart of patient inclusion. ERUS: endorectal ultrasound; MRI: magnetic resonance imaging; ADC: apparent diffusion coefficient; * diffusion-weighted imaging was performed in all patients, but four had a hip implant causing artefacts.

A total of 222 patients were referred for primary T category assessment, of which 86 were included (see flow chart in Figure 1). The mean age was 70 years, with ages ranging from 37 to 98. There were 51 males and 35 females. Histopathology showed 62 adenomas and 24 adenocarcinomas, of which 11 were T1, and 13 were T2. In two patients, the deep resection margin could not be assessed with certainty after endoscopic resection, but they abstained from completion resection because of old age. Both were followed up endoscopically for at least six months without any sign of recurrence and were classified as pT1. The ERUS procedure was performed by one of five radiologists, of whom two accounted for 62% of the examinations, i.e., 29 and 24 procedures, while the remaining three performed between 10 and 13 each. All patients underwent ERUS and SWE. Eighty-three patients underwent an MRI, while three were omitted due to claustrophobia or a pacemaker implant. In 4 of the 83 patients, ADC values were not obtained due to hip arthroplasty implant artefacts.

### 3.2. Endorectal Ultrasound

ERUS, as seen in Table 2, yielded sensitivity, specificity, PPV, NPV, and accuracy of 0.79, 0.95, 0.86, 0.92, and 0.86, respectively, for discrimination between adenoma and early rectal cancer. There was a substantial agreement between T category assessed by ERUS and histopathology with a kappa of 0.675.

**Figure 2:**
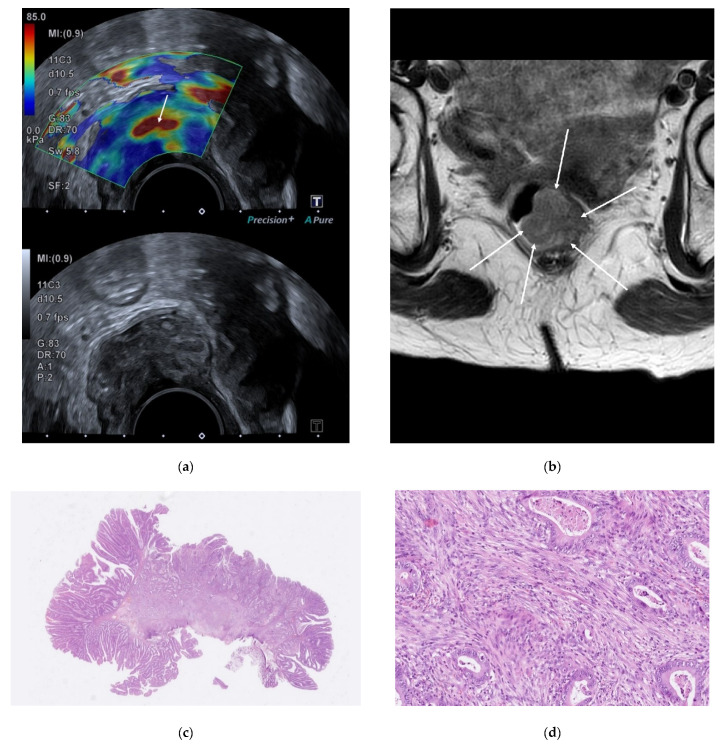
A 93-year-old female patient. (**a**) Endorectal ultrasound with shear-wave elastography color map on the upper image and the corresponding B-mode on the lower image. Within the lesion is a red (hard) area. (**b**) Magnetic resonance T2-weighted image of the lesion. The white arrows indicate the lesion boundaries. (**c**) Histopathology revealing an adenomatous polyp with a central focus of adenocarcinoma. (**d**) 200× magnification shows an SF of 70%.

**Figure 3:**
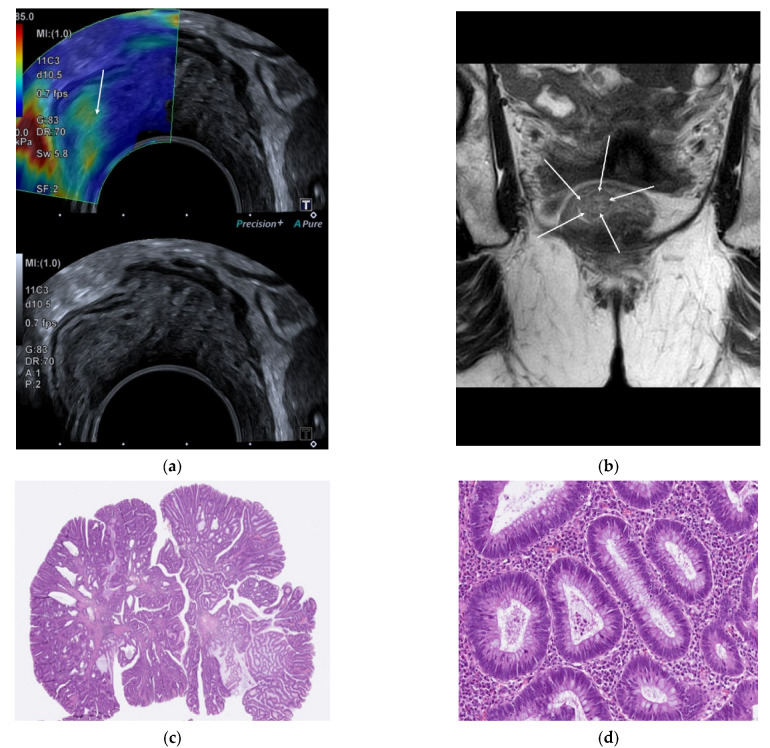
An 81-year-old female. (**a**) Endorectal ultrasound with shear-wave elastography color map superimposed on a B-mode image on the upper image and the corresponding B-mode on the lower image. The lesion is primarily blue and green, indicating a soft tumor. (**b**) Magnetic resonance T2-weighted image of the lesion. The white arrows indicate the lesion boundaries. (**c**) Histopathology revealing an adenoma. (**d**) 200× magnification shows an SF of <10%.

Of the 62 adenomas, 59 (95%) were correctly classified, and 3 were overstaged, of which 2 were classified as uT1 and 1 as uT2. Of the 11 pT1 tumors, 6 (55%) were correctly classified, and 5 were understaged as adenomas. Of the 13 pT2 tumors, 9 (69%) were correctly classified, 3 were understaged as uT1, and 1 was overstaged as uT3. If the two elderly patients desisting from completion surgery were excluded due to uncertain pT category, then ERUS correctly classified 5/9 pT1 tumors (55%) and understaged 4/9. Figure 2 and Figure 3 show images of ERUS, SWE, MRI, and histopathology.

### 3.3. Magnetic Resonance Imaging

MRI showed sensitivity, specificity, PPV, NPV, and accuracy of 0.73, 0.90, 0.73, 0.90, and 0.78, respectively, for discrimination between adenoma and adenocarcinoma. There was a moderate agreement between T category assessed by MRI and histopathology with a kappa of 0.499. Of the 61 (90%) adenomas, 55 (90%) were correctly classified, and 6 were overstaged, 4 as mrT1 and 2 as mrT2. Of the 10 pT1 tumors, 5 (50%) were correctly classified, and 5 were understaged as adenomas. Of the 12 pT2 tumors, 5 (42%) were correctly classified, and 2 were understaged, 1 as mrT1 and 1 as an adenoma, and 5 were overstaged, 4 as mrT3 and 1 as mrT4. One of the two elderly patients who refrained from completion surgery did not have an MRI because of claustrophobia. Excluding the other as well led to 4/9 (44%) pT1 tumors being correctly classified by MRI.

### 3.4. Shear-Wave Elastography and Diffusion-Weighted Imaging

**Figure 4:**
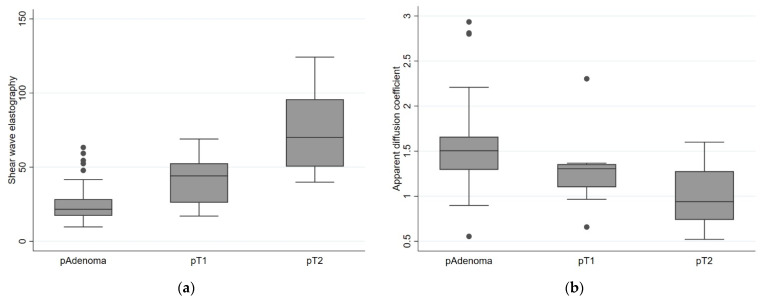
Box plot showing means with percentiles and outliers for pAdenomas, pT1, and pT2 tumors of: (**a**) Shear-wave elastography in kilopascal (kPa) with a difference between the means with a chi^2^ of 16.15 and a *p*-value of <0.001; (**b**) apparent diffusion coefficient in 10^−3^ mm^2^/s with a chi^2^ of 1.29 and a *p*-value of 0.525.

The mean SWE value increased with the presence of malignancy, with an increasing pT category, whereas the ADC value decreased, as seen in Table 3 and Figure 4.

**Figure 5:**
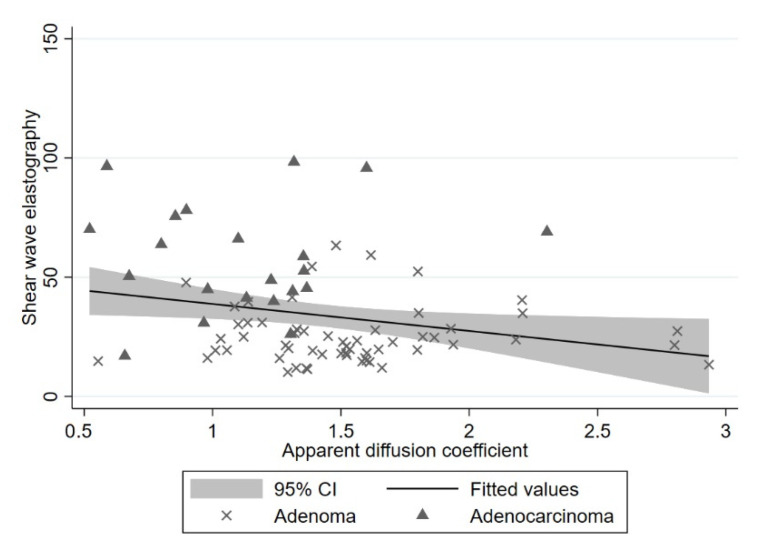
Correlation between shear-wave elastography (SWE) in kPa and apparent diffusion coefficient (ADC) in 10^−3^ mm^2^/s. There is a negative correlation with a *p*-value of 0.025. Each point is plotted with the SWE value and the corresponding ADC value. X represents an adenoma, and a triangle represents an adenocarcinoma. The line represents the fitted values, and the grey area represents the 95% confidence interval.

The mean of the SWE value was 24.87 (95% CI 21.86–27.88), 40.99 (95% CI 30.42–51.56), and 72.12 (95% CI 57.84–86.41) for adenoma, pT1, and pT2, respectively. One-way ANOVA showed a significant difference between the groups (i.e., adenoma, pT1, and pT2) with a *p*-value of <0.001. For ADC the mean value was 1.534 (95% CI 1.417–1.650), 1.288 (95% CI 0.993–1.583), and 0.997 (95%CI 0.804–1.189) for adenoma, pT1, and pT2, respectively. One-way ANOVA showed no difference in the mean of ADC values between the groups with a *p*-value of 0.525, but additional Jonckheere-Terpstra tests showed highly significant (*p* < 0.0001) trends towards increasing SWE and decreasing ADC across the groups. We found a negative correlation between SWE and ADC with a *p*-value of 0.025, since tumors with an increase in SWE showed a decrease in ADC value, shown in Figure 5. Excluding the two elderly patients refraining from completion surgery changed the mean pT1 SWE and ADC values only slightly: to 39.19 ± 17.20 and 1.312 ± 0.469, respectively.

The ROC curve analysis of SWE showed an area under the curve of 0.8831. We selected three cut-off values with sensitivity, specificity, PPV, NPV, and accuracy, ranging from 0.75 to 0.83, 0.82 to 0.90, 0.63 to 0.75, 0.90 to 0.91, and 0.83 to 0.86, respectively. Details are presented in Table 2.

### 3.5. Stromal Fraction

**Figure 6:**
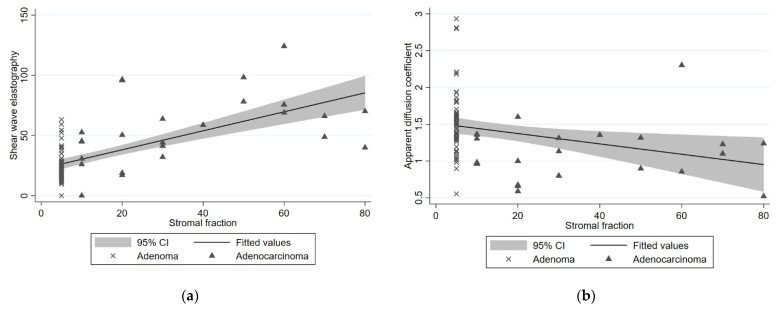
Plots showing the correlation between stromal fraction (SF) and shear-wave elastography (SWE) and between SF and apparent diffusion coefficient (ADC). Each point is plotted as the percentage SF and the corresponding value of SWE or ADC. The line represents the fitted values, and the grey area represents the 95% confidence interval. (**a**) Correlation between SF and mean SWE values in kPa. There is a positive correlation with a *p*-value of <0.001. (**b**) Correlation between SF and ADC values in 10^−3^ mm^2^/s. There is a negative correlation with a *p*-value of 0.011.

All adenomas had an SF of less than 10%. The SF score for adenocarcinomas ranged from 10% to 80%. A total of 8 of 24 cancers showed a high SF, of which 2 were pT1 tumors, and 6 were pT2 tumors. As shown in Figure 6, we found a positive correlation between SF and SWE with a *p*-value of <0.001 and a negative correlation between SF and ADC with a *p*-value of 0.011.

## 4. Discussion

The differentiation between adenoma and early rectal cancer is a challenging yet important task, as the primary tumor assessment forms the basis for treatment decisions at multidisciplinary team meetings [18]. Our results showed that SWE can be used as a relevant addition to the ERUS procedure to differentiate between adenoma and early rectal cancer with an NPV of 0.92. Our results are similar to those found in other studies where SE was used [9,10]. This suggests that SWE and SE both perform quite well, although a direct comparison has not yet been performed. Interobserver variability among different operators is low in both SWE and SE [19,20]. We found that a cut-off value of 40 kPa yielded the highest accuracy for discrimination between an adenoma and early rectal cancer, but a different ultrasound machine, software version, or transducer may yield a different cut-off value. The ERUS T category assessment also performed well, and it is important to keep in mind that this study was performed in a clinical setting where the operators were not blinded; therefore, the T stage assessment, as well as the SWE measurement, may be subject to bias. We also showed that ERUS performed with a slightly higher accuracy than MRI in patients with early rectal cancer, which is similar to previous findings [21]; however, MRI is superior in advanced rectal cancer and provides specific imaging features related to tumor aggressiveness such as lymph node staging, MR circumferential resection margin involvement, and extramural vascular invasion [22]. It may be noted that MRI overstaged 13% of tumors (11/83), including 10% of adenomas, whereas ERUS only overstaged 5% of the tumors (4/86), including 5% of the adenomas (3/62). From a clinical point of view, overstaging may have far more serious consequences for the patient than understaging. If a rectal tumor is judged as benign or very superficial, a local resection will usually be attempted first (endoscopic or transanal) and only followed by major surgery if unsuccessful. It is well established that a previous attempt at local resection has no adverse effects on the outcome of a subsequent salvage operation [23,24,25]. ERUS as a supplement to MRI in imaging of early rectal tumors is, therefore, strongly recommended. We investigated DWI but found no difference between the means of the three groups; however, there was a tendency for the ADC value to decrease from adenoma to pT1 and from pT1 to pT2. Even though we found no significant correlation between the ADC value and pT stage, DWI provides eye-catching imaging features which helps radiologists to detect rectal tumors and suspicious lymph nodes, and it is likely to predict response to chemoradiotherapy [26]. The negative correlation between SWE and ADC has previously been reported in advanced rectal cancer [12]. The endoscopic ability to distinguish between adenomas and adenocarcinomas prior to imaging assessment may be improved. A recent study using real-time near-infrared confocal laser endoscopy showed interesting results, and perhaps with this technique, we will be able to detect cancer at a cellular level in the future [27].

We found a positive correlation between SWE and SF, and tumors with a high SF (i.e., above 50%) had higher SWE values compared to those with low SF. Tumors with high SWE values are likely to have a poorer prognosis in terms of both overall survival and disease-free survival than those with lower SWE values [14]. However, we should keep in mind that the majority of our data are derived from benign adenomas. The relationship between tumor SF and UE has been investigated in other organs. In murine breast cancer, SWE was found to be correlated with tumor fibrosis [28], and SWE was also found to be significantly correlated with SF in peritumoral stromal tissue of benign and malignant breast tumors [29]. Another study, however, found no correlation between SWE and SF in invasive ductal carcinoma [30]. In locally advanced pancreatic cancer, a correlation between SE and stromal proportion was found [31]. As to ADC values, we found a negative correlation with SF with a *p*-value of 0.011. Previous investigations have shown varying results. Two studies investigated the ability of ADC to predict a high tumor–stroma ratio. Both studies included all T categories, but a majority were of advanced stage. One study [32] found a significant correlation, whereas the other study [33] found no correlation. These studies differ from ours on several parameters. Our population consists of both adenomas and T1 and T2 categories of adenocarcinomas. The majority of our study population had adenomas, whereas the other two studies were mainly of advanced cases. Furthermore, we evaluated the correlation between the absolute SF and ADC, whereas the other studies evaluated the ability to predict a high tumor-stroma ratio.

The limitations of our study are mainly related to the fact that it was performed in a real-life clinical setting. It was a single-center study with a small study population, and the majority of tumors were benign adenomas. The ERUS procedures were performed by one of five radiologists. All were experienced ERUS examiners; however, each had an individual preferred method of approach. Applying SWE measurements was sometimes challenging as this was a new methodology introduced in our clinic during the COVID-19 pandemic. Four of the radiologists were dedicated to the gastrointestinal and abdominal area and performed the MRI evaluations. The radiologists were not blinded to MRI or ERUS findings when assessing either modality. Other challenges were posed by the COVID-19 pandemic with its restrictions on hospital staffing or by the patients themselves. For example, two patients were very elderly and refrained from a completion resection which would otherwise have been advisable due to histopathological risk factors. Excluding these two individuals from relevant analyses changed our results only negligibly. It is important to note that our study does not pretend to be able to predict the definitive treatment of the patient (which is based on histopathology and other information not obtainable by imaging) but only to guide the initial treatment choices. On the other hand, the real-life setting of our study may be regarded as a strength since it may increase its generalizability.

Finally, we used an endocavity probe with a forward-pointing convex array. When assessing occluding tumors, using the forward-looking probe is advantageous, as it is independent of whether the tumor is passable or not; however, when assessing adenomas and small tumors, using a rotating probe with 3D reconstruction may improve the assessment of the T category.

## 5. Conclusions

Complex rectal adenoma and early rectal cancer can be assigned to T categories using both ERUS and MRI, but in our study, ERUS classified more adenomas correctly. Shear-wave elastography showed a high ability to distinguish between adenoma and early rectal cancer. Increased SF correlated with an increase in SWE and a decrease in ADC value.

## Figures and Tables

**Table 1:** Magnetic resonance imaging scanning protocol parameters.

Sequences	T2-Weighted Sagittal	T2-Weighted Transversal	T2-Weighted Coronal	T2-Weighted Transversal	Diffusion-Weighted
Coil	anterior	anterior	anterior	anterior	anterior
TR (µs)	3000	3000	3000	3000	3500
TE (µs)	90	90	90	90	93
Slice thickness (mm)	3	5	4	3	5
Gap (mm)	0.3	0.5	0.4	0.3	0
FOV (mm^2^)	270 × 270	270 × 270	270 × 270	270 × 270	160 × 120
Voxel	0.7 × 0.7	0.7 × 0.7	0.7 × 0.7	0.7 × 0.7	2.5 × 2.5
Matrix	512	880	880	640	256
Flip angle (°)	90	90	90	90	90
B-values	-	-	-	-	0, 300, 500, 800, 1000

TR: repetition time; TE: echo time; FOV: field of view.

**Table 2:** Performance of endorectal ultrasound, magnetic resonance imaging, and shear-wave elastography for discrimination between adenoma and early rectal cancer.

Adenoma vs. pT1/pT2	Sensitivity (95% CI)	Specificity (95% CI)	PPV (95% CI)	NPV (95% CI)	Accuracy	Kappa
**ERUS**	0.79 (0.58–0.93)	0.95 (0.87–0.99)	0.86 (0.65–0.97)	0.92 (0.83–0.97)	0.86	0.675
**MRI**	0.73 (0.50–0.89)	0.90 (0.80–0.96)	0.73 (0.50–0.89)	0.90 (0.80–0.96)	0.78	0.499
**SWE Cut-off**						
**≥** **32.1**	0.83	0.82	0.63	0.91	0.83	
**≥** **39.9**	0.79	0.89	0.73	0.92	0.86	
**≥** **41.3**	0.75	0.90	0.75	0.90	0.86	

ERUS: endorectal ultrasound; MRI: magnetic resonance imaging; SWE: shear-wave elastography; vs.: versus; CI: confidence interval; PPV: positive predictive value; NPV: negative predictive value.

**Table 3:** Mean values of shear-wave elastography (kilopascal) and apparent diffusion coefficient (10^−3^ mm^2^/s) of pAdenomas, pT1, and pT2 adenocarcinomas.

	Mean SWE ± SD	95% CI	Range	Mean ADC ± SD	95% CI	Range
pAdenoma	24.87 ± 11.92	21.86–27.88	9.7–63.3	1.534 ± 0.46	1.417–1.650	0.554–2.934
pT1	40.99 ± 17.63	30.42–51.56	17.0–69.0	1.288 ± 0.44	0.993–1.583	0.658–2.303
pT2	72.12 ± 25.91	57.84–86.41	39.9–124.2	0.997 ± 0.34	0.804–1.189	0.521–1.600

SWE: shear-wave elastography; SD: standard deviation; CI: confidence interval; ADC: apparent diffusion coefficient.

## Data Availability

Data available on request due to restrictions, e.g., privacy or ethical. The data presented in this study are available on request from the corresponding author. The data are not publicly available due to the fact that they contain personal records.
